# Near peer integrated teaching for final year medical students

**DOI:** 10.1007/s40037-016-0255-7

**Published:** 2016-02-23

**Authors:** Laurence Fulford, Victoria Gunn, Gregory Davies, Claire Evans, Tanzeem Raza, Michael Vassallo

**Affiliations:** Royal Bournemouth Hospital, Castle Lane East, Bournemouth, Dorset, UK; Poole General Hospital, Longfleet Road, Poole, Dorset, UK; University of Bournemouth, Fern Barrow, Poole, Dorset, UK

**Keywords:** Peer teaching, Integrated, Medical students

## Abstract

**Introduction:**

Medical students preparing for final exams need practical and theoretical knowledge. We evaluated a junior doctor led integrated programme delivering theoretical and practical teaching.

**Methods:**

An 8-week junior-doctor led teaching programme was set up for final year medical students. Theory, OSCE and bedside teaching on selected weekly clinical themes were run. Satisfaction was evaluated using a questionnaire survey.

**Results:**

Almost all agreed that the programme was useful and that an integrated approach to teaching was more beneficial than separate and unrelated lectures and practical teaching. The majority agreed that theory sessions and practical sessions had improved their confidence for finals and agreed they felt more prepared for work as a doctor. Most agreed that the Facebook® group provided an easily accessible platform for communication and sharing learning resources. Some comments, however, highlighted limitations particularly in the ability to answer difficult questions.

**Conclusion:**

Integrated teaching by junior doctors in small groups appeared to be an efficient teaching method (for theoretical and clinical skills) for medical students, improving their confidence for finals and life as a doctor and provided useful opportunities for junior doctors to develop as clinical teachers. This can be a useful blueprint for other hospitals.

## Introduction

Final year medical students need to acquire practical and theoretical knowledge to sit finals and start work. Teaching programmes addressing this must consider the complex context of adult learning that requires a variety of teaching methods [1, 2]. Factors we considered included reduced attention span and need for multiple teaching methods. By linking theory to practical sessions it is possible to consolidate knowledge using different learning styles to suit individual learners. Developing such a programme also offers opportunities to develop skills and practices amongst clinical teachers as highlighted by the United Kingdom (UK) General Medical Council (GMC) [3]. In the UK newly qualified doctors are enrolled into a two-year foundation programme. They are expected to develop as clinical teachers in accordance with the GMC guidance and standards set in the foundation curriculum [4]. To help achieve these standards we developed an integrated teaching programme for final year students preparing for finals. It also aimed to provide pastoral support during their clinical attachments and opportunities for junior doctors to develop teaching skills and demonstrate educational leadership.

## Methods

**Fig. 1:**
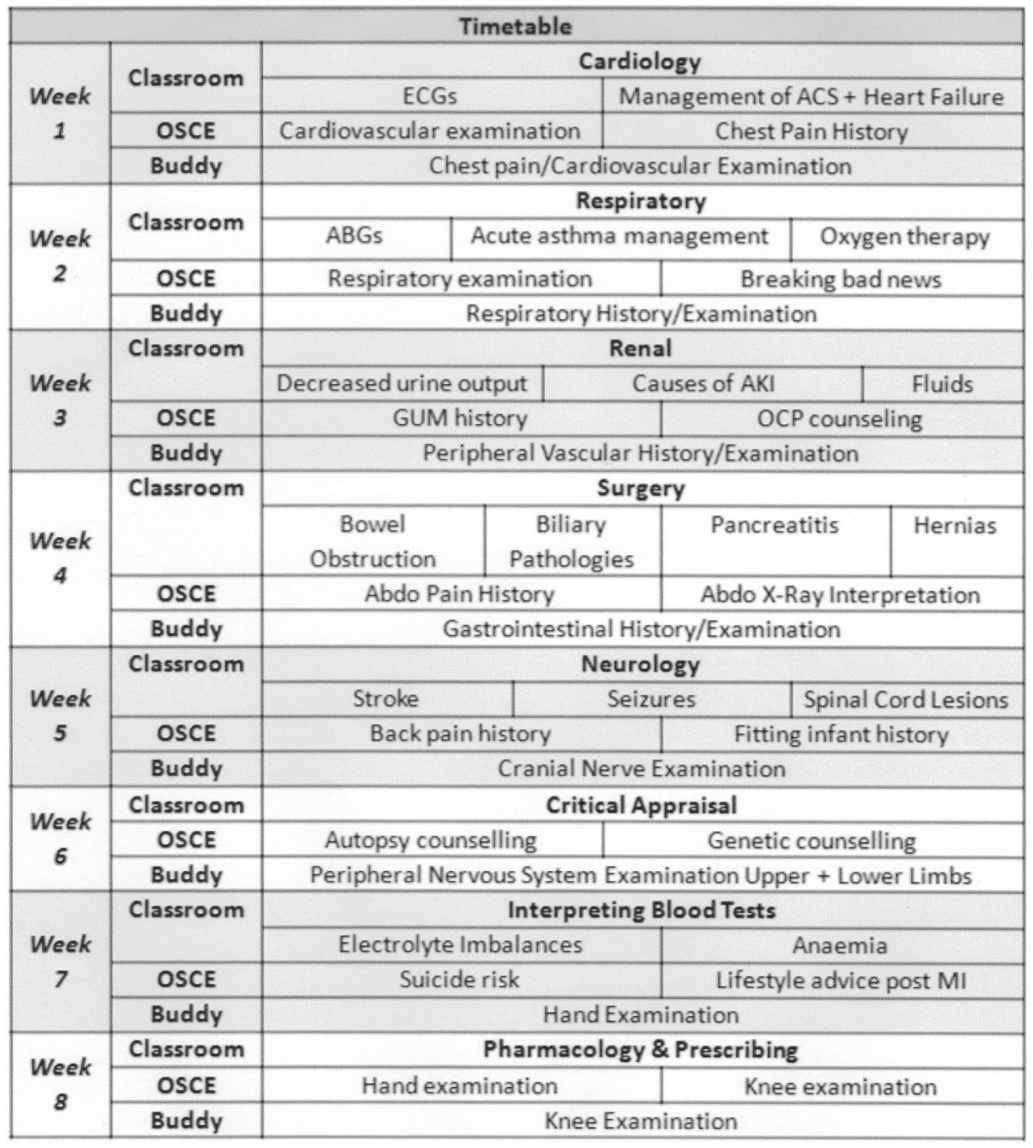
Timetable of the programme highlighting the integration of different teaching styles

Final year medical students were assigned to Bournemouth and Poole Hospitals for 8-week attachments. The programme had eight themes on key medical emergencies and subjects often encountered in finals. These were taught in three styles of sessions (Fig. 1): The level of the content was tracked to the level required by Foundation Year 1 curriculum.

- Classroom teaching focused on key facts, ways of remembering them, passing finals and being a safe doctor. Each hour session was split into 20–30 min blocks run by different tutors to provide variety and help maintain focus.
- Objective structured clinical examination (OSCE): In each hour session, two common stations were taught in small groups. Stations addressed physical examination skills and communication scenarios. Students learned by observing, practising and receiving feedback in a supportive environment.
- Buddy scheme: Each student was paired with a doctor for one-to-one bedside teaching aimed to recognize clinical signs, develop practical skills and provide pastoral support.

Junior doctors signed up to specific sessions. They were expected to have passed OSCE stations themselves before being allowed to teach and to individually prepare for the sessions. Clear learning objectives and session resources were provided to enhance preparation and performance. All trainees had to undertake a ‘Developing the Clinical Teacher’ supervised learning event as a curriculum competency with a supervisor of their choice [4]. This tool was used to support the development of a doctor’s skill in teaching and/or making presentations. Post session feedback from attendees was offered as an opportunity to reflect on teaching practice. Social media was used to promote participation by establishing a Facebook® group facilitating communication between teachers and students about teaching times, venues, resources and feedback. The course was evaluated in two ways. Firstly, feedback was collected at the end of a sample of classroom sessions based on the two questions (i) how useful was the teaching? and (ii) was the teaching pitched at the right level? At the end of the programme participants were also invited to fill in a detailed questionnaire. These was evaluated using a 5-point Likert scale and free text comments.

The project was reviewed by the local Research & Development department. We obtained confirmation that the project and data collection met criteria for a service evaluation rather than research as it involved evaluation of existing teaching sessions with questionnaires completed voluntarily and anonymously. This work did not require submission for review by ethics nor National Health Service permission. The work was carried out in accordance with the Declaration of Helsinki in particular anonymity was guaranteed and consent for evaluation was obtained.

## Results

Thirty-four from a total of 38 (89 %) participated. All rated the programme as useful and 34/34(100 %) felt it was pitched at the right level. 23/38(60 %) participated in a more detailed questionnaire and provided comments. 22/23(96 %) attended theory and 21/23(91 %) attended OSCE sessions. All agreed that a programme of this style should be available to all final medical students and all agreed that an integrated approach is more beneficial at their stage than separate unconnected lectures and practical teaching. The majority felt that theory 20/23(87 %) and practical sessions 21/23(91 %) improved their confidence for the written and OSCE element of finals. 19/23(83 %) agreed that near peer teaching was more relevant and focused than sessions provided by more senior clinicians and 21/23(91 %) agreed that they felt more prepared for work as a doctor. 18/23(78 %) used the Facebook® group but 20/23(87 %) agreed that this was an easily accessible platform for communication and sharing resources. Feedback for the buddy programme was mixed with only 11/23(48 %) responses. Only 2/11(22 %) agreed that the buddy improved confidence for final year examinations and 5/11(45 %) were undecided. Pass rates for final year students attending the hospitals before the teaching programme were above 95 % and remained so for students undergoing the teaching.

Free comments were thankful and complimentary of the programme and the doctors who taught them. They reported it to be ‘concise’, ‘well-organized’ and ‘relevant’. Students found teachers ‘approachable’ and ‘relatable’ and mentioned that OSCE practice with senior doctors can be ‘more intimidating’. Some comments, however, highlighted the limitations of near peer teachers stating, ‘when asked difficult questions they are less able to answer’. Teachers themselves found involvement beneficial to their development, improving teaching, organizational and leadership skills. The majority volunteered to teach in subsequent groups.

## Discussion

Integrated learning for finals was well received. All students agreed this style of teaching on a chosen weekly subject in a variety of contexts (classroom, OSCE and buddy) was an effective way of teaching, increasing confidence and should be available to all final medical students. Arguably an important factor contributing to the success was the attention to set up the programme bearing in mind factors that facilitate adult learning. It is well established that the focused learning time for an adult is 20 min. Without a suitable break, attention fluctuates down to three-four minute spurts. Altering lecture content in short classroom sessions and offering a variety of teaching environments may have contributed to maintaining attention in participants, maximizing retention of information and improving satisfaction [1, 2]. The use of social media made learning resources more accessible and encouraged communication between tutors and students. This supports evidence that social media can be an important collaborative learning tool [5]. A poorly performing aspect was the uptake, by both tutors and students, of the buddy scheme. It was intended that the buddy could provide more of pastoral support and mentoring on an individual basis. Feedback suggested that poor uptake was due to time limitations on the foundation trainees who had work commitments as well as their own training requirements. This is reflected in students being undecided as to whether the buddy scheme has improved their confidence. A more proactive selective process with pairing of students with the same tutors for the sessions and probably more importantly providing dedicated time for communication and teaching time-tabled within the programme may have led to increased uptake.

A strength of the programme was the peer and near peer teaching. Such teaching has been proven to be effective and an enjoyable way of providing information, and trainers can develop as role models [6, 7] In our case students agreed that the programme provided more relevant and focused teaching than sessions provided by more senior healthcare professionals and has helped them feel more prepared to start work as a foundation doctor. This may have been because they felt more at ease being taught by the near peers, benefitting from personal anecdotes and tips that newly qualified doctors gave based on their recent experience of starting work. There was recognition, however, that junior doctors were less able to answer difficult questions than senior colleagues when asked. In such an eventuality the teacher could discuss the answer with the senior clinicians supporting the programme or outside the programme by asking clinical and educational supervisors. Although this was a limitation, as the knowledge of the teacher is a very important aspect of learning, it was felt that knowing the limits of one’s own knowledge and acknowledging it is an important part of development as a teacher. In addition the skill required in identifying physical signs needs very well trained staff to see the minor and major flaws in performance of the students. Although teachers would have passed OSCE stations themselves and were expected to volunteer for sessions they prepared for, there was no substantial external senior scrutiny of this. An area for future improvement is that of more robust quality assurance of the programme.

Feedback from the junior doctor teachers confirmed that this programme was a good opportunity to develop competences as clinical teachers and pass on their recent experiences of final year examinations. It also offered experience in educational leadership and management. Overall the programme was a positive experience for both students and junior doctor teachers and may serve as a model for other organizations hosting students and junior doctors.
